# The immediate effects of a single autogenic drainage session on ventilatory mechanics in adult subjects with cystic fibrosis

**DOI:** 10.1371/journal.pone.0195154

**Published:** 2018-03-29

**Authors:** Elliot Wallaert, Thierry Perez, Anne Prevotat, Gregory Reychler, Benoit Wallaert, Olivier Le Rouzic

**Affiliations:** 1 Faculté des Sciences de la Motricité, Université Catholique de Louvain, Louvain-la-Neuve, Belgium; 2 CHU-Lille, Centre de Ressource et de Compétence pour la Mucoviscidose, Service de Pneumologie et Immuno-allergologie, Hôpital Calmette and Univ. Lille, Lille, France; 3 Service de Médecine Physique et Réadaptation, Cliniques Universitaires Saint-Luc and Institut de Recherche Expérimentale et Clinique (IREC), Pôle de Pneumologie, ORL et Dermatologie, Université Catholique de Louvain, Brussels, Belgium; University of Cape Town, SOUTH AFRICA

## Abstract

**Introduction:**

The aim of this study was to gain insight into the physiological changes occurring in subjects with cystic fibrosis (CF) after autogenic drainage (AD). Changes in respiratory system resistance (Rrs), reactance (Xrs), and spirometry were analyzed in adult CF subjects after a single AD physiotherapy session.

**Methods:**

This prospective observational study was conducted during the annual check-up of adult CF subjects in stable condition. Spirometry and Rrs and Xrs measurements using the forced oscillations technique at 5, 11, and 19 hertz (Hz) were performed before and 30 min after a 20-min AD session. Control CF subjects were tested at baseline and 50 min without AD. Results are expressed as mean ± standard deviation or median [interquartile range].

**Results:**

Thirty subjects were included in the physiotherapy group (age 29 [25–34] years, forced expiratory volume in 1 s (FEV_1_) 40.3 [30.1–57.9]% predicted) and 11 in the control group (age 31 [28.5–36.5] years, FEV_1_ 43.6 [31.1–51.9] % predicted). No significant changes in any parameter were observed in the control group. AD modestly but significantly increased the forced vital capacity (FVC) and FEV_1_ (p<0.001). Inspiratory resistance was also significantly improved by AD: Rrs_5_ from 5.74±2.39 to 5.24±2.17 cmH_2_O/L/s, p<0.05; Rrs_11_ from 4.83±1.98 to 4.32±1.7 cmH_2_O/L/s, p = 0.003; and Rrs_19_ from 4.18 [3.46–5.07] to 3.86 [2.76–4.98] cmH_2_O/L/s, p<0.001. In contrast, AD had no significant effects on frequency dependence of resistance (Rrs_5_–Rrs_19_) or expiratory resistance. Inspiratory Xrs_5_, but not ΔXrs_5_ (expiratory—inspiratory Xrs), was improved by AD (p<0.05). Moderate correlations were detected between the improvement in FEV_1_ and FVC and inspiratory resistance (r = 0.53, p = 0.005 and r = 0.44, p = 0.02, respectively).

**Conclusion:**

A single session of AD improved inspiratory airway resistance, except in the distal airways. The forced oscillations technique provides a new tool for understanding the pathophysiological effects of airway clearance physiotherapy in CF.

## Introduction

Cystic fibrosis (CF) is the most common genetic disease in the Caucasian population. Cause of death is linked to respiratory disease due to the accumulation of mucus in the airways, which ultimately leads to secondary infections, bronchiectasis, and respiratory failure [1].

Physiotherapy is an integral part of the treatment of CF [2] and the clinical efficacy of airway clearance is well established [3][4][5][6]. According to a recent Cochrane systematic review, no single airway clearance technique has shown superiority [7]. However, patients usually express a strong preference for self-administered airway clearance techniques, such as autogenic drainage (AD) or the active cycle of breathing technique, rather than postural drainage and percussion [3][6]. There is an urgent need to gain insight into the nature and underlying mechanisms of the physiological changes occurring in CF subjects after physiotherapy [8]. The forced oscillations technique (FOT), developed in the 1950s by DuBois [9], allows analysis of ventilatory mechanics, resistance (Rrs), and reactance (Xrs), both globally and at different levels of the respiratory tract during tidal breathing [10]. A previous study of FOT showed that adult CF subjects had higher resistance and reactance values compared with the healthy population, suggesting that the FOT might provide sensitive indicators of respiratory system resistive and reactance properties in CF subjects [11]. Recently, Guimaraes et al. showed improvements in resistance and conductance in CF subjects, as measured by plethysmography, following respiratory physiotherapy by slow and complete expiration in the lateral posture with opened glottis (ELTGOL) or Flutter interventions [12].

Since the FOT has been shown to provide relevant information on respiratory mechanics, the primary aim of this study was to gain insight into the physiological changes occurring in CF subjects after physiotherapy with AD, rather than to demonstrate clinical efficacy of AD *per se*. To this end, we assessed the impact of a single AD session on Rrs and Xrs via FOT measurements in stable CF subjects.

## Materials and methods

### Subjects

Consecutive adult subjects with CF followed at the Cystic Fibrosis Center (Centre de Ressource et de Compétence pour la Mucoviscidose) in Lille were enrolled during their annual check-ups. The diagnosis of CF was based on the presence of (1) a sweat chloride concentration >60 mmol/L and/or genetic confirmation of two CF-associated mutations and (2) two clinical features consistent with CF [13]. All subjects were clinically stable (no antibiotics for an exacerbation in the preceding 4 weeks). Patients who had received lung transplants or had confirmed asthma were excluded from the study. The study protocol was approved by the appropriate ethics committee (N°17/068, Lyon). All individuals gave written informed consent. Approval for the use of these data was provided by the Institutional Review Board of the French Learned Society for Pulmonology (CEPRO 2015–004).

### Study design

All tests were performed in a single visit. The subjects did not perform physical therapy on the morning of the tests and received no bronchodilator or mucolytic treatment in the 12 h preceding the tests. For the physiotherapy group, FOT and spirometry were performed before the 20-min AD session (baseline) and after a 30-min rest period following completion of the AD session. The tests were performed in the same order at both time points. To avoid a possible effect of the first spirometry forced expiratory maneuvers on the second FOT measurements, a non-randomized control group of 11 CF subjects underwent two sets of tests (the same tests in the same order) 50 min apart, but did not receive physiotherapy.

### Autogenic drainage

AD is an airway clearance technique developed by Dab [14] and Chevallier [15]. The technique is based on non-forced expiration during controlled breathing at different levels of vital capacity. While sitting, the patient first performs diaphragmatic breathing at low lung volume following a cycle of (i) slow inspiration through the nose, (ii) pause for 3 s, (iii) non-forced exhalation in a sighing manner through the nose or mouth, and (iv) repetition of steps (i) to (iii) at the same lung volume until mucus movement can be felt. The entire breathing cycle is repeated at progressively increasing lung volumes, so that mucus is moved from the small to the mid-sized to the large airways. Finally, the mucus is evacuated from the trachea by huffing [16]. In this study, the cycles were repeated for a total AD session of ~20 min.

### Forced oscillations technique

FOT was performed with a Resmon Pro FULL (Restech; Milan, Italy) in multifrequency mode at 5, 11, and 19 hertz (Hz). The subjects used a nose clip and mouthpiece that stabilized the tongue position as they effortlessly breathed in their tidal volume. The test was done with the patient in a sitting position and the cheeks firmly supported to reduce airway shunting. Before each subject was tested, machine calibration was checked using the calibration test object provided by the manufacturer. The test was performed in accordance with the European Respiratory Society Task Force recommendations on forced oscillation testing, and acceptability was confirmed on-site in accordance with the criteria proposed by the task force [17]. We measured whole-breath and partitioned (inspiratory and expiratory) values for resistance at 5, 11, and 19 Hz (Rrs_5_, Rrs_11_, and Rrs_19_, respectively) and for reactance at 5 Hz (Xrs_5_) [18]. The difference between inspiratory Rrs_5_ and Rrs_19_ was considered representative of the small airway contribution, and ΔXrs_5_ (Xrs expiratory—Xrs inspiratory) was considered an index of expiratory flow limitation (EFL) using a cut-off value of 1 cm H_2_O [18]. Three valid measurements were taken, and the mean of the three measurements are reported. Results are expressed as absolute values (cmH_2_O/L/s) [19].

### Spirometry

Spirometry was performed using the MICRO spirometer 5000 (Medisoft; Sorinnes, Belgium). Forced vital capacity (FVC), forced expiratory volume in 1 s (FEV_1_), FEV_1_/FVC, and forced expiratory flow between 25 and 75% of the FVC (FEF_25–75_) were measured. Baseline spirometry results are expressed as z-scores, in accordance with the recent multiethnic Global Lung Function Initiative spirometry equations [20]. By analogy with COPD and in the absence of specific data for CF, a threshold improvement of 100 mL FEV_1_ was considered clinically significant [21].

### Sputum

All sputum produced during each 20-min AD session and for 30 min after was collected in a sterile, pre-weighed container (balance precision 1.0 mg). After weighing, the wet secretions were heated in an oven at 60°C for 72 h and then re-weighed to obtain the dry weight.

### Statistical analysis

Statistical analysis was performed with R software (version 3.4.2). The Shapiro—Wilks W test was used to determine whether the data were normally distributed. Quantitative continuous variables are presented as the median and interquartile range (25^th^–75^th^ percentile) or mean ± standard deviation, and categorical variables as the number and percentage. Student’s t-test was used to analyze normally distributed. Wilcoxon test and Kuskal-wallis to analyze non-normal data. Pearson’s r test was used to determine correlations between the quantitative parameters. The power calculation for this study was based on data published for CF patients [12]. Using a two-sided alpha of 0.05 and a reduction of ≥0.5 cmH_2_O/L/s for Rrs_5_ as statistically significant, a minimum of 36 subjects was required to provide a power of 0.8. P<0.05 was considered statistically significant.

## Results

Thirty-six subjects were enrolled in the physiotherapy study, of whom six were excluded due to an inability to obtain valid breathing cycles during FOT testing. A final total of 30 CF subjects in the physiotherapy group (14 women and 16 men) and 11 CF subjects in the control group (7 women and 4 men) were included. Their characteristics are summarized in Table 1.

**Table 1:** Characteristics of the cystic fibrosis patients.

Variable		CF controls (N = 11)	Physiotherapy group (N = 30)
Age	years	31.0 [28.5–36.5]	29.0 [25.0–34.0]
Weight	kg	54.0 [49.0–59.0]	52.5 [49.2–60.5]
Height	cm	161.2 ± 9.5	165.4 ± 9.0
BMI	kg/m^2^	20.6 [19.7–22.9]	19.9 [18.6–21.0]
FEV_1_	mL	1310 [965–1760]	1350 [1068–2143]
	% predicted	43.6 [31.1–51.9]	40.3 [30.1–57.9]
	z-score	−4.37 [−4.90 - −3.69]	−4.71 [−5.39 - −3.28]
FVC	mL	1980 [1610–2870]	2420 [2030–3813]
	% predicted	61.7 [52.6–68.3]	62.8 [48.1–79.9]
	z-score	−2.90 [−3.31 - −2.53]	−3.20 [−4.42 - −1.56]
FEV_1_/FVC	%	59.4 [49.6–67.1]	54.2 [50.7–63.2]
	% predicted	70.5 [58.0–78.7]	65.2 [59.9–77.1]
	z-score	−3.03 [−3.72 - −2.40]	−3.51 [−3.76 - −2.67]
FEF_25–75_	mL/s	610 [410–880]	545 [447–1115]
	% predicted	20.1 [12.9–27.3]	15.4 [11.0–27.6]
	z-score	−4.43 [−4.92 - −3.35]	−4.63 [−5.28 - −3.52]
Homozygous ΔF508		6 (55%)	15 (50%)
Heterozygous ΔF508		5 (45%)	8 (26.7%)
Other mutation		0 (0%)	7 (23.3%)
*Pseudomonas* colonization		9 (82%)	25 (83.3%)
Other colonization		1 (16.7%)	5 (16.7%)
EPI		11 (100%)	28 (93.3%)
Diabetes		2 (18%)	8 (26.7%)

Results are expressed as median [interquartile range] or mean ± SD according to the Shapiro—Wilk test results for quantitative data, or as number (%) for frequencies. No significant differences were detected between the two groups for any parameter. BMI, body mass index; EPI, exocrine pancreatic insufficiency; FVC, forced vital capacity; FEV, forced expiratory volume in one second; FEF_25–75_, 25–75% forced expiratory flow.

The spirometry, Rrs, and Xrs results are presented in Table 2. All subjects exhibited moderate to severe obstructive ventilatory disorder characterized by reduced FEV_1_ and FEV_1_/FVC, increased bronchial resistance, and decreased reactance (Table 1). There were no significant differences between the two groups when comparing baseline values. The control group showed no significant changes in any parameters between the baseline and second evaluation (Table 2).

**Table 2:** Changes in pulmonary function tests and resistance measures in the physiotherapy and control groups.

	CF controls (N = 11)			Physiotherapy group (N = 30)		
Variable	Baseline	Second value	Δ	Baseline	After AD	Δ
FVC (mL)	1980 [1610–2870]	2050 [1725–2760]	50 [-10–105]	2420 [2030–3813]	2445 *** [2195–4043]	115# [67–200]
FEV_1_ (mL)	1310 [965–1760]	1250 [955–1735]	10 [-25–50]	1350 [1068–2143]	1320 *** [1108–2175]	45 [37.5–95]
FEV_1_/FVC (%)	59.4 [49.6–67.1]	59.0 [49.7–65.6]	−0.4 [−1.0–0.3]	54.2 [50.7–63.2]	53.7 [50.6–62.4]	0.28 [−1.3–0.7]
FEF_25–75_ (mL/s)	610 [410–880]	660 [380–885]	0 [-15–105]	540 [450–985]	545 [447–1115]	10 [-17.5–30]
Rrs_5_ inspiratory	5.14 ± 1.93	5.19 ± 2.04	0.05 ± 0.59	5.74 ± 2.39	5.24 ± 2.17 *	−0.5 ± 1.13
Rrs_5_ expiratory	5.90 ± 2.07	5.98 ± 2.38	0.07 ± 0.50	6.66 ± 2.80	6.40 ± 2.85	−0.27 ± 1.30
Rrs_5_ whole-breath	5.58 ± 1.91	5.66 ± 2.14	0.08 ± 0.51	6.29 ± 2.58	5.94 ± 2.54 *	−0.35 ± 1.08
Rrs_11_ inspiratory	4.42 ± 1.41	4.54 ± 1.52	0.12 ± 0.38	4.83 ± 1.98	4.32 ± 1.70 **	−0.51 ± 0.87 ^#^
Rrs_11_ expiratory	5.14 [3.98–5.58]	5.13 [3.99–5.80]	0.16 [−0.19–0.35]	5.28 [3.84–6.45]	4.77 [3.57–7.19]	−0.14 [−0.85–0.35]
Rrs_11_ whole-breath	4.73 ± 1.45	4.9 ± 1.66	0.17 ± 0.51	5.33 ± 2.19	4.99 ± 1.98 *	−0.35 ± 0.86
Rrs_19_ inspiratory	3.52 [2.76–4.27]	3.45 [3.03–4.25]	−0.07 [−0.18–0.26]	4.18 [3.46–5.07]	3.86 *** [2.76–4.98]	−0.42 ^#^ [−1 - −0.01]
Rrs_19_ expiratory	3.88 [3.23–4.55]	3.95 [3.44–4.61]	−0.07 [−0.30–0.26]	4.48 [3.54–5.58]	4.17 [3.31–6.28]	−0.14 [−0.58–0.24]
Rrs_19_ whole-breath	4.02 [2.98–4.23]	3.90 [3.33–3.98]	−0.07 [−0.28–0.24]	4.43 [3.49–5.34]	4.02 * [3.14–5.70]	−0.27 [−0.69–0.11]
Rrs_5_–Rrs_19_ inspiratory	1.47 [0.51–2.19]	0.86 [0.32–2.12]	−0.2 [−0.34–0.31]	0.77 [0.34–2.1]	1.00 [0.43–2.17]	−0.05 [−0.58–0.33]
Xrs_5_ inspiratory	−2.64 [−2.91- −1.73]	−2.50 [−3.08-−1.23]	0.31 [−0.38–0.61]	−1.86 [−3.47 - −0.94]	−1.77 * [−3.06 - −0.92]	0.14 [−0.09–0.49]
Xrs_5_ expiratory	−2.80 [−4.70 - −1.94]	−2.93 [−5.33 - −1.83]	0.03 [−0.87–0.05]	−2.87 [−4.08 - −1.45]	−2.70 [−4.18 - −1.15]	0.06 [−0.18–0.33]
Xrs_5_ whole-breath	−2.65 [−3.92 - −1.83]	−2.79 [−4.40 - −1.50]	−0.12 [−0.73–0.27]	−2.61 [−3.84 - −1.18]	−2.23 [−3.72 - −1.11]	0.06 [−0.10–0.32]
ΔXrs	0.68 [−0.27–1.91]	0.76 [0.31–2.30]	0.41 [0.32–0.81]	0.46 [−0.01–1.68]	0.82 [0.23–1.50]	0.07 [−0.29–0.61]

Tests were performed at baseline and after 50 min for the control group or at baseline and after 20 min AD + 30 min rest (50 min total) for the physiotherapy group. Resistance and reactance are expressed as cmH_2_O/L/s. Δ, mean ± SD or median [interquartile range] for the change between second value and baseline. FVC, forced vital capacity; FEV, forced expiratory volume in one second; FEF_25–75_, 25–75% forced expiratory flow; Rrs_5_, Rrs_11_, and Rrs_19_, respiratory system resistance at 5, 11, and 19 Hz, respectively; Rrs_5_–Rrs_19_, difference between resistance at 5 and 19 Hz; Xrs, respiratory system reactance at 5 Hz; ΔXrs, Xrs expiratory—Xrs inspiratory. Comparison with baseline values:

*p<0.05;

**p = 0.003;

***p<0.001.

Comparison with control group:

^#^p<0.05.

**Fig 1:**
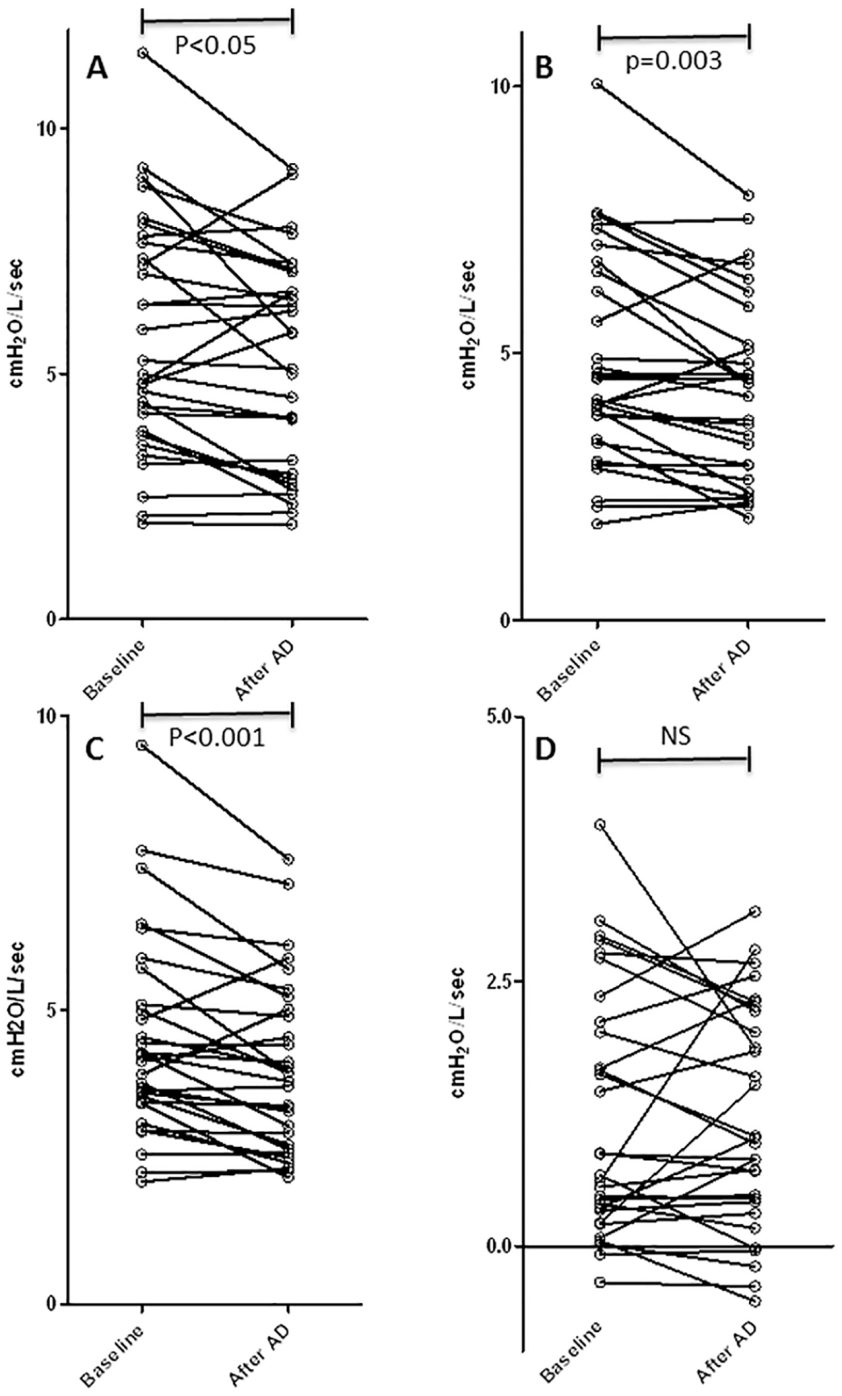
Individual values of inspiratory resistances before and after autogenic drainage in 30 CF patients. A, Rrs_5;_ B, Rrs_11;_ C, Rrs_19;_ D, Rrs_5_–Rrs_19_.

The physiotherapy group patients underwent an AD session lasting an average of 20 min. The mean sputum wet and dry weights produced between the first and second measures (obtained in 27/30 subjects) were 11.235 g ± 7.53 and 0.687 g ± 0.56, respectively. After AD, there were modest but significant improvements in FVC and FEV_1_ (both p<0.001; Table 2). FEV_1_ was improved by at least 100 mL in 7 patients (23.3%) in the physiotherapy group and 2 (18.1%) in the control group (not significant). No change was observed in FEF_25–75_ after AD. We observed a similar magnitude of improvement in inspiratory Rrs_5_, Rrs_11_, and Rrs_19_ after AD. Total (whole-breath) resistance at 11 and 19 Hz were also significantly decreased after AD. The change in inspiratory Rrs_11_ and Rrs_19_ in the AD group was statistically significant when compared with the control group (p<0.05). Inspiratory Rrs_5_ decreased by at least 0.5 cmH_2_O/L/s in 13 subjects in the AD group (Fig 1) and 1 subject in the control group (p = 0.06). There was no significant change in Rrs_5_–Rrs_19_ after AD. The improvements in inspiratory resistance were not significantly different at 5, 11, and 19 Hz. AD had no significant effect on expiratory resistance. Inspiratory Xrs_5_ was improved after AD (p<0.05); however, no other significant changes in Xrs were observed.

ΔXrs_5_ at baseline was high (>1 cmH_2_O/L/s), consistent with a significant EFL in 10 patients (33.3%) in the physiotherapy group and 4 (36%) in the control group (not significant), but this was not improved by AD. There were no correlations between the sputum (wet or dry) weight and the change in any functional parameter measured. Moderate to good correlations were observed between the change in inspiratory resistance and improvement of both FEV_1_ (r = 0.53, p = 0.005) and FVC (r = 0.44, p = 0.02).

## Discussion

Our study is the first to measure the effects of AD airway clearance on respiratory mechanics in CF subjects using FOT. The results show: (1) a moderate decrease in inspiratory resistance in the global (Rrs_5_) and proximal (Rrs_11_ and Rrs_19_) airways, but not in the distal compartment (Rrs_5_–Rrs_19_); (2) a statistically (but not clinically) significant improvement in FEV_1_ and FVC but not in FEF_25–75_; and (3) a correlation between improved resistance and the improvement in FVC and FEV_1_.

The baseline resistance and reactance values were elevated in the CF subjects, these results are similar to those reported by Lima et al. in adult CF subjects [11]. To our knowledge, there has been only one study to date examining the effects of a chest physiotherapy session (Flutter vs ELTGOL) on airway resistance in CF subjects that demonstrated a significant improvement in plethysmographic resistance [12]. Our results are in agreement with those post-ELTGOL values. However, plethysmography is not able to distinguish between the distal or proximal origin of measured resistance. Our results using FOT showed decreased inspiratory Rrs_5_, Rrs_11_, and Rrs_19_, but not Rrs_5_–Rrs_19_. According to Brashier and Salvi, Rrs_5_, Rrs_19_, and Rrs_5_–Rrs_19_ represent resistance of the global airways, the large airways, and the small airways, respectively [22]. The lack of improvement in Rrs_5_–Rrs_19_ observed in our study is consistent with the lack of change in FEF_25–75_. Taken together, these results suggest that AD does not significantly increase the luminal caliber of the distal airways, at least after a single session. This may be because of the timing of measurements. We could argue that the sputum coming from the proximal part of the lung is significantly cleared immediately after the physiotherapy session, in contrast to the more peripheral secretions. In addition, we cannot exclude the possibility that AD might induce a slight post-exercise bronchoconstriction. The speed of the airflow in distal airways, and thus the ability to move secretions, is low; therefore, it could take longer to expel mucus from the respiratory tract, or AD may simply not be effective on this portion of the bronchial tree. An improvement in Rrs_5_–Rrs_19_ may not be detectable within 30 min after AD and merits further investigation. In our subjects, global expiratory resistance did not significantly decrease after AD. The reason for this is not clear, but reflex adduction of the glottis during expiration could interfere with the measurement of expiratory resistance [23][24]. Alternatively, marked airway instability during expiration in CF patients could compromise the reproducibility of FOT measurements in the expiratory phase, and thus blunt any significant change after a single AD session. In COPD and asthma, inspiratory Rrs has slightly better between-day reproducibility than does total Rrs [25]. To our knowledge, there are currently no data on the reproducibility of various FOT parameters in CF subjects.

The mechanism underlying the improvement in Xrs_5_ is complex and not completely understood. Low frequency reactance reflects the elastic properties of the lung (elastance) and is indicative of opening of small airways and collapsed areas of the lung [26]. The reduction in negative inspiratory Xrs_5_ values (i.e., increased reactance) and the observed improvement in FVC are consistent with the hypothesis that AD improves airway permeability, decreases gas trapping, and thus improves distensibility of the small airways [27]. A significant proportion of our subjects exhibited EFL at baseline according to ΔXrs. In COPD subjects, ΔXrs was shown to be a reliable indicator of EFL compared with the reference technique using esophageal pressure [28]. The same concept most likely applies to CF subjects. AD had no impact on this parameter, suggesting either a predominant proximal effect of the technique or a major contribution of small airway structural remodeling to EFL. Measurement of resistance by forced oscillations has also been studied in non-CF subjects with bronchiectasis. Our results are in agreement with those studies, which demonstrated a significant decrease in global resistance (Rrs_5_) and no change in Xrs_5_ after respiratory therapy [29][30]. We can hypothesize that the global resistance is partly related to a contribution of sputum to luminal airway occlusion. Several studies have also emphasized the potential for FOT to enable better understanding of the complex pathophysiology of obstructive lung diseases such as asthma [31] and COPD [10].

The short-term effects of airway clearance on FEV_1_ and FVC in our study are discordant with the results of other studies using various maneuvers. Thus, no significant change in FEV_1_ and FVC was observed with the ELTGOL technique [12], whereas improvements were seen after supervised chest physiotherapy with a positive expiratory pressure (PEP) mask [32] or an incentive spirometer, but not after AD [33]. In our study, although the FEV_1_ and FVC improvements were statistically significant, they may be irrelevant in clinical practice based on a minimal clinically important difference (MCID) of 100 mL for FEV_1_ [21]. Only 23% of subjects in our study showed improvement in FEV_1_ that exceeded 100 mL. Evaluation of the short-term clinical benefit of AD on patient-reported outcomes was beyond the scope of this study. Although the use of a PEP mask has been shown to significantly improve FEV_1_ and FVC in CF subjects [32], albeit below the 100 mL MCID, those subjects received salbutamol prior to airway clearance. Indeed, most studies include a salbutamol dose 20 min before physiotherapy [12][30][32]. We chose not to administer a bronchodilator to avoid potential bias. However, this choice is questionable because, as mentioned, bronchodilators are routinely used before daily physical therapy and some CF subjects do find this effective [16][34].

Although we avoided any potential effects of spirometry on FOT measurements by performing the baseline FOT first, the results of our control group confirm that changes in resistance were not a consequence of the first spirometry. We chose to perform measurements at 30 min after the AD session based on the results of a lung clearance index study showing that the effect of airway clearance in CF subjects was maximal at 30 min compared with 5 min, 1 h, or 2 h [35].

## Conclusion

Our study shows that a single session of AD improved airway inspiratory resistance, except in the distal airways. The FOT opens up new avenues of mechanistic research to understand the pathophysiological effects of airway clearance physiotherapy in CF. Similar studies with other airway clearance techniques or exercise, and evaluation of longer treatment periods, would improve our understanding of the magnitude and range of parameters affected by the different techniques.

## Supporting information

S1 Table Raw data for CF controls and CF patients. (XLSX)
